# Design of a Reverse Electrodialysis Plant for Salinity Gradient Energy Extraction in a Coastal Wastewater Treatment Plant

**DOI:** 10.3390/membranes13060546

**Published:** 2023-05-24

**Authors:** Tamara Sampedro, Carolina Tristán, Lucía Gómez-Coma, Marcos Fallanza, Inmaculada Ortiz, Raquel Ibañez

**Affiliations:** Departamento de Ingenierías Química y Biomolecular, Universidad de Cantabria, Av. Los Castros 46, 39005 Santander, Spain; sampedrot@unican.es (T.S.);

**Keywords:** reverse electrodialysis, energy recovery, water reclamation, techno-economic assessment, Levelized Cost of Energy

## Abstract

The chemical potential difference at the discharge points of coastal Wastewater Treatment Plants (WWTPs) uncovers the opportunity to harness renewable salinity gradient energy (SGE). This work performs an upscaling assessment of reverse electrodialysis (RED) for SGE harvesting of two selected WWTPs located in Europe, quantified in terms of net present value (NPV). For that purpose, a design tool based on an optimization model formulated as a Generalized Disjunctive Program previously developed by the research group has been applied. The industrial scale-up of SGE-RED has already proven to be technically and economically feasible in the Ierapetra medium-sized plant (Greece), mainly due to a greater volumetric flow and a warmer temperature. At the current price of electricity in Greece and the up-to-date market cost of membranes of 10 EUR/m^2^, the NPV of an optimized RED plant in Ierapetra would amount to EUR117 thousand operating with 30 RUs in winter and EUR 157 thousand for 32 RUs in summer, harnessing 10.43 kW and 11.96 kW of SGE for the winter and summer seasons, respectively. However, in the Comillas facility (Spain), this could be cost-competitive with conventional alternatives, namely coal or nuclear power, under certain conditions such as lower capital expenses due to affordable membrane commercialization (4 EUR/m^2^). Bringing the membrane price down to 4 EUR/m^2^ would place the SGE-RED’s Levelized Cost of Energy in the range of 83 EUR/MWh to 106 EUR/MWh, similar to renewable sources such as solar PV residential rooftops.

## 1. Introduction

The crash in the gas supply system of the EU promoted by the Ukraine war, in addition to the current climate urgency, has led to a scenario of energy uncertainty [1,2]. In this context, the last Conference of the Parties (COP 26, celebrated in Glasgow, Scotland) [3] claimed that carbon neutrality would be reached by 2050. Thus, the development and exploitation of new renewable sources are nowadays hotspots instead of traditional obstacles produced by high investment costs [4]. This issue will translate into lessening dependence on fossil fuels and, consequently, a reduction in the environmental impacts derived from their use, in addition to greater world economic stability [5].

On the other hand, the overexploitation of renewable natural water resources, together with damage to water bodies due to human activities and serious worldwide droughts as a consequence of climate change [6], is leading society to turn to the use of non-conventional water sources, such as by water reclamation and desalination [7]. Therefore, new water sources must be developed alongside traditional ones to try to meet the water needs of the population. In this sense, the EU is focusing on developing specific legislation to promote water reclamation and its subsequent reuse for non-potable uses. Consequently, intensifying the water–energy nexus could be driven by integrating energy generation technologies with water reclamation processes [8].

Against this backdrop, salinity gradient energy (SGE), also known as blue energy, is an emerging emission-free energy based on the physicochemical potential existing when two water streams of different salinity concentrations are mixed [9,10,11,12]. Among the benefits of SGE, compared to more widespread renewable energies such as photovoltaic or wind energy, it stands out that it is generated continuously without restrictions due to seasonal variations. Moreover, this type of energy could represent a niche opportunity for the self-production and sustainable energy supply of different water treatment facilities since the standard technologies employed to extract this energy do not cause any water damage [13]. Thus, and promoting the principles of the circular economy increasingly encouraged by European Union policies, the effluents generated in coastal urban wastewater treatment plants (WWTPs) and desalination facilities represent a potential source of SGE extraction in combination with waters of different salinity [14]. In the case of WWTPs, which are necessary to control water pollution and prevent direct discharge into natural water bodies, the effluents could be used for water street cleaning, industrial processes, or agriculture [15].

In the European Union alone, 29.2 billion m^3^ of treated municipal wastewater is generated annually [16], which in most cases is discharged into the environment despite it being a region suffering from water scarcity. Water reclamation is penalized by the additional energy consumption of more stringent regeneration treatments. The specific energy (kWh/m^3^) of wastewater treatment is intensified, the higher the quality of effluent demanded [17].

One of the technologies with the best prospectives for extracting energy from SGE is reverse electrodialysis (RED), an electrochemical process based on the transfer of ions through positively and negatively charged membranes [18,19,20]. This technology has key components such as the type of membrane chosen, profiled or not profiled; the use (or not) of woven spacers or the number of cell pairs assembled; and critical variables such as temperature, water composition, and flow rate [21,22]. Nowadays, the main efforts in this research field are focused on developing membranes with better properties to maximize power density, expressed in W/m^2^ [23]. In this regard, it is essential to reduce electrical resistance by modifying the thickness of membranes and to facilitate the ionic flux of key monovalent ions (Na^+^ and Cl^−^) by increasing permselectivity towards them [24]. Furthermore, the development of RED membrane modules at an affordable cost is one of the major challenges for its large-scale implementation.

The latest scientific publications have demonstrated a continuous increment in the interest in harnessing energy from the chemical composition of wastewater streams. Recently, Gómez-Coma et al. (2020) [25] proposed the use of SGE given by effluent and seawater in coastal WWTPs for water reclamation boosting. In a laboratory plant, they managed to generate a gross power of 1.43 W/m^2^ (55 Wh/m^3^) operating continuously for 480 h, with no fouling or power decay problems. So far, the main efforts have been focused on the development of predictive models such as the semi-empirical model proposed by Hossen et al. (2020) [26], the mathematical model described by Ortiz-Martínez et al. (2019) [27] to study the influence of different variables, a study of the presence of divalent ions by Pintossi et al. (2021) [28], or the coupling of a one-dimensional model with CFD analysis by La Cerva et al. (2017) [29].

However, to advance from the current Technology Readiness Level (TRL 5, demonstration plant) of the integrated SGE-RED systems to TRL 6, corresponding to an industrial pilot, more powerful decision-making tools are needed. In this sense, full-scale RED deployment would require a techno-economic assessment including environmental parameters that consider the whole process design and operational decision gap from the RED stack to the full system.

Tristán et al. (2023) [30] developed an optimization model formulated as a Generalized Disjunctive Programming (GDP) problem that incorporates a RED stack mathematical model from our research group. This decision-making tool is able to define the cost-optimal process design. The solution to the GDP problem provides the optimal plant topology and RED units for stated working conditions, maximizing the net present value (NPV).

The objective of this work is to determine the optimal topological design of a large-scale RED plant according to the maximum NPV. The optimum is determined as a function of (1) site-specific operating conditions (effluent temperature and flow rate) and (2) economic parameters (membrane and electricity price).

For this purpose, we apply Tristán et al.’s optimization model to two representative case studies in the EU where a RED plant recovers energy from low-salinity effluent from coastal UWWTPs mixed with a high-salinity water source such as seawater. Therefore, it is intended to evaluate the economic feasibility of scaling up the process from laboratory and pilot plant scale to an industrial facility with a system configuration that maximizes the net present value of the RED process.

## 2. Materials and Methods

### 2.1. Definition of the WWTP Sites Addressed

An evaluation of the profitability and feasibility of the application of RED to extract SGE will be conducted for the following deployment sites:

(1) S1, WWTP of Comillas, located in the North of Spain (ES). This facility has a treatment capacity of 35,200 population equivalent (p.e.), and it is located in the coastal area of the municipality of Comillas, next to the Cantabrian Sea.
(2) S2, WWTP of Ierapetra, located in Greece (GR). This medium-sized plant has a treatment capacity of 25,700 p.e. and is located on the warm coast of the Mediterranean Sea.

The choice of these two specific cases has been made in order to make a comparison of the techno-economic feasibility between WWTPs under changeable weather conditions and located in countries with different energy grid mixes.

The temperature fluctuations between summer and winter significantly affect the efficiency of the RED system; the decrease in temperature is detrimental to the power output [26]. In addition, rainfall and the increase in population during the tourist peak season could result in high variability of the wastewater flow throughout the year. S1 is particularly strongly affected by rainfall patterns. Based on these premises, two weather operating conditions have been approached and are described in Table 1 for both sites: (1) winter season and (2) summer season.

The amount of energy in the design extracted by the RED electrochemical process between two water streams is determined by the salt concentration gradient. Both plants, S1 and S2, are located on the coastline, thus presenting access to seawater as a high-salinity stream. Typical salinity in the Mediterranean Sea is 0.59 M NaCl (Ierapetra WWTP), and in the Atlantic Ocean it is 0.51 M NaCl (Comillas WWTP), as reported in the literature [31,32]. The concentration gradient in this work is defined by seawater in contact with a wastewater treatment plant effluent with an optimum concentration of 0.015 M NaCl for maximum power density achievement (fixed value for all study scenarios) [27].

### 2.2. Techno-Economic Assessment

This section summarizes the main equations and assumptions of the optimization model developed by Tristán et al. (2023), which are detailed in depth in their publication of Computers and Chemical Engineering [30]. The optimization model is formulated as a Generalized Disjunctive Programming (GDP) problem that incorporates a finite-difference RED stack model from our research group to define the cost-optimal process design. The background of the mathematical model has been described in previous publications [25,27,33,34,35].

The solution to the optimization problem provides: (i) the hydraulic arrangement; (ii) the flows and concentrations of all feasible streams present in the RED plant; and (iii) the working conditions of the active RED units that maximize the net present value (NPV) of the RED process for the two given site-specific conditions.

The superstructure considers all alternatives for a given number of RED candidate units and yields all feasible flowsheet designs for the RED process, from which an optimization algorithm will derive the optimal solution.

The objective function f(x) of Tristán et al.’s (2023) [30] model maximizes the net present value (NPV, in EUR) of the RED plant, as given in Equation (1):

(1) $$NPV=\frac{TNP\cdot LF\cdot 8760\left(ep+cp\cdot ef\right)-TAC}{CRF}$$

where TNP is the total net power (kW), LF is the load factor or the actual working hours of the RED plant (as % of the total hours in a year), ep is the electricity price (EUR/kWh), cp is the carbon price (EUR/tCO_2e_), ef is the emission factor (expressed in kg CO_2e_/kWh), and CRF is the capital recovery factor (−). Disbursement flows involve the incurred annualized capital and yearly operational expenses that yield the total annual cost (TAC in EUR/year, defined by Equation (2)) of the RED process.

(2) TAC=CRF·CAPEX+OPEX

The capital costs (CAPEX) comprise the RED module, pumps, and civil and infrastructure costs; and the annual operating costs (OPEX) include the electricity cost from pumps, the replacement cost of membranes, and operation and maintenance (O&M) labor costs. The reciprocal of the capital recovery factor, CRF (−), converts the TAC and yearly benefits into their respective present values, and is calculated through Equation (3).

(3) $$CRF=\frac{r}{1-{\left(1+r\right)}^{-LT}}$$

where r is the interest rate (expressed in %) and LT is the expected plant lifetime, stated in years. The total net power (${NP}_{r}$ in kW) is defined as the sum of the net power produced by each active RED unit (rϵRU):

(4) $$TNP=\sum_{r\epsilon RU}NP_{r}$$

Table 2 summarizes the financial conditions assumed for RED process optimization for the given sites and RED stack parameters. The characteristics of the typical commercial RED modules available are listed in Table 3. We set national and European electricity prices for cases S1 and S2. We also quantify the environmental benefits of RED technology integration based on the abated greenhouse gas emissions (GHG), the emissions factor of each specific country’s energy mix, and the emission allowance price in the EU emissions trading system. Given the great influence of IEM development and linked cost reduction on RED economic viability on small and large scales, we evaluate two membrane prices reflecting the lowest cost reported in the literature [36] (10 EUR/m^2^) and economies-of-scale cost reduction (4 EUR/m^2^) [37]. An expected IEM lifetime (LT_m_) of 15 years and an expected plant lifetime of 30 years have been assumed based on RED upscaling literature studies [38].

An evaluation of the influence of grid electricity rates, consumable equipment costs, and operating conditions (temperature and LC flow rate) in the WWTPs of Comillas and Ierapetra results in the optimization of 12 different case studies.

The overall efficiency of the energy harvesting stage in each case study is measured by the Total Net Specific Energy (TNSE) given by Equation (5) and expressed in kWh/m^3^.

(5) $$TNSE=\frac{TNP}{Q_{LC}}$$

where $Q_{LC}$ is the low-concentrated feed stream flow rate to the RED plant (m^3^/h). In addition, the model provides data on the Levelized Cost of Energy (LCOE) which makes it possible to compare in economic terms the competitiveness in the energy market of an emerging renewable technology such as SGE. The LCOE has been calculated according to the definition in Equation (6).

(6) $$LCOE=\frac{CRF\cdot CAPEX+OPEX}{TNP\cdot 8760\cdot LF}$$

## 3. Results and Discussion

A detailed analysis of the results has been carried out, taking into account the economic feasibility through NPV, on the one hand, and looking at the distribution of the annualized costs in specific cases and the optimal modular and hydraulic configuration, on the other hand.

### 3.1. NPV Optimization

The results of the cases under study to optimize the net present value are presented in Table 4, taking into account the following parameters: (i) seasonal conditions, (ii) membrane price, (iii) electricity cost of the associated grid mix, and (iv) size of the WWTP. The information derived from the SGE-RED process simulation and optimization software addresses the number of RED units (RU), NPV, TNP, and TNSE as a function of the volume of treated wastewater passing through the membrane modules.

Looking at the cost–benefit ratio over the entire lifetime of the plant monetized to date, it is apparent that the technology is cost-effective in all cases analyzed in site S2 for the specified operating conditions. In S1 the implementation of the SGE-RED technology at current membrane price conditions, around 10 EUR/m^2^, would not even meet the return on investment (break-even point). In this regard, the optimization simulations have determined that the extraction of SGE using RED under up-to-date conditions of development and commercialization of RED equipment (S1—case 2 and S1—case 4) is unfeasible, due to the non-profitability of the project at this site in both the winter and summer seasons. The results obtained in S1 reaffirm that the technical feasibility is strongly conditioned by the operating conditions. Nonetheless, S1 has a preliminary foresight of positive profitability.

However, reaching a cost-competitive price of 4 EUR/m^2^ would result in a positive monetary balance in case studies S1—case 1 and S1—case 3, which would vary in the range of EUR 66.9 × 10^3^–EUR 55.5 × 10^3^, respectively. In this future case simulated from the global optimization of the technology, the cost-effectively generated energy could reach 7.1 kW during the winter season and would be around 5.6 kW for the summer period. Notwithstanding the colder water temperature in the winter season, S1—case 1 has a better energy production ratio due to the greater availability of water and, therefore, the feasibility of installing a larger number of RUs. In the summer season there is a lower effluent flow, probably due to the absence of rainfall during the analyzed period, which in fact leads to an operational limitation.

Considering S2, the results are more promising. Even though the net benefits vary due to O&M and consumable costs, the integration of SGE-RED is economically feasible in all case studies approached. The simulated data, as expected, suggest that a slightly higher effluent flow rate in contact with Mediterranean seawater on the Greek coast (warmer than the Atlantic Ocean) improves the exploitation of SGE and provides a better economic balance.

In the second scenario, the HC concentration (0.59 M) is slightly higher than S1 (0.51 M), as is the operating temperature considered in the stacks (18–24 °C). In addition, the volumetric flow of reclaimed water is more abundant in S2. These three factors converge in a substantial intensification of the performance of RED in the extraction of useful energy for a defined NaCl concentration gradient, expressed in power density, W/m^2^. In this regard, the resulting optimized design is mainly characterized by an increase in the predicted number of operating units.

In view of the seasonal periods, the operation of the modules in conditions closer to the optimum of extractable SGE (summer season) enables us to increase the size of the industrial installation (>RUs) with an economic balance with larger benefits. Taking as references, S2—case 5 (winter) and S2—case 9 (summer) with NPV EUR 320 × 10^3^ and EUR 361 × 10^3^, respectively, for an equal optimum of RUs, the NPV is appreciably better in the warmer season with a power growth from 14.45 kW to 15.57 kW. The results obtained with a membrane price of 4 EUR/m^2^ seem to be attainable, in line with previous studies in which a break-even price for the IEMs of 4.3 EUR/m^2^ was determined [38].

For an accurate analysis of the operation of the RED technology subjected to seasonal variations, it is necessary to look at the overall period of operation (winter + summer). The specific ratio of winter/summer RUs for the global case studies is defined as follows:

- S1 − ES − 4 EUR/m^2^ − winter (case 1)/summer (case 3) = 29/21 RUs
- S2 − GR − 4 EUR/m^2^ − winter (case 5)/summer (case 9) = 58/58 RUs
- S2 − GR − 10 EUR/m^2^ − winter (case 6)/summer (case 10) = 30/32 RUs
- S2 − EU − 4 EUR/m^2^ − winter (case 7)/summer (case 11) = 53/54 RUs
- S2 − EU − 10 EUR/m^2^ − winter (case 8)/summer (case 12) = 22/24 RUs

This ratio is close to 1 in all assumptions, and therefore, there is no oversizing of RUs in the design of the SGE-RED plants. However, depending on the operating profitability and following the optimization results of this study, in each period of time only that number of RED units determined as optimal would operate.

**Figure 1:**
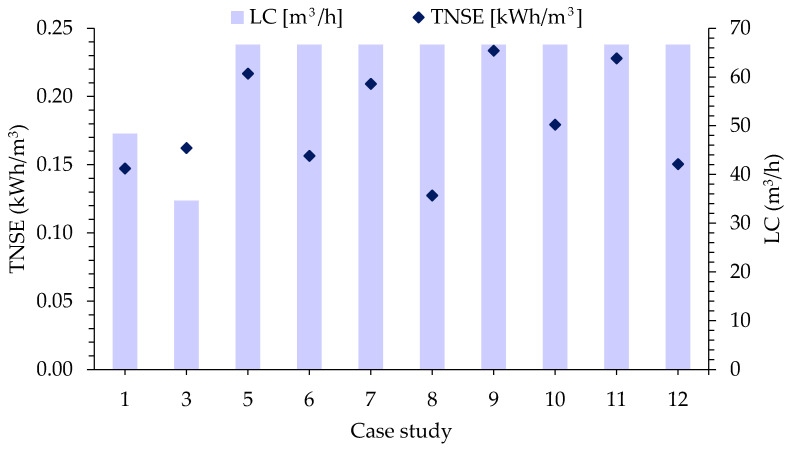
Treated water flow per branch of RED units and specific energy produced for each respective case. LC: low-concentrated stream (treated wastewater flow rate) and TNSE: Total Net Specific Energy measured in kWh/m^3^ of LC entering RED stack. WWTP of Comillas: S1—cases 1 and 3 and WWTP of Ierapetra: S2—cases 5–12.

In Figure 1 the LC flow rate for each parallel RU branch has been plotted together with SGE extraction performance (TNSE), using TNSE as an indicator of energy efficiency in SGE harnessing. Following this statement, 0.162 kWh/m^3^ could be reached at Comillas through the implementation of recirculation, while at Ierapetra, with more LC feed availability, the specific energy harvested would be up to 0.234 kWh/m^3^ of effluent entering the RED modules.

### 3.2. Optimal RU Configuration

The modelling and optimization of the RED produces the optimal hydraulic configuration of membrane modules, determining the low-concentrated (LC, reclaimed water) and high-concentrated (HC, seawater) water flows for each module, the recirculation flow rate if applicable, and the route from beginning to end RED units through which they run. In addition, it defines the variation of the saline gradient for all the hydraulic lines (inflow, intermediate flows, and outflow). The extended results are presented below for S2—case 8 corresponding to the winter season, referring to the average European electricity price and for a cost of 10 EUR/m^2^ of membrane.

**Figure 2:**
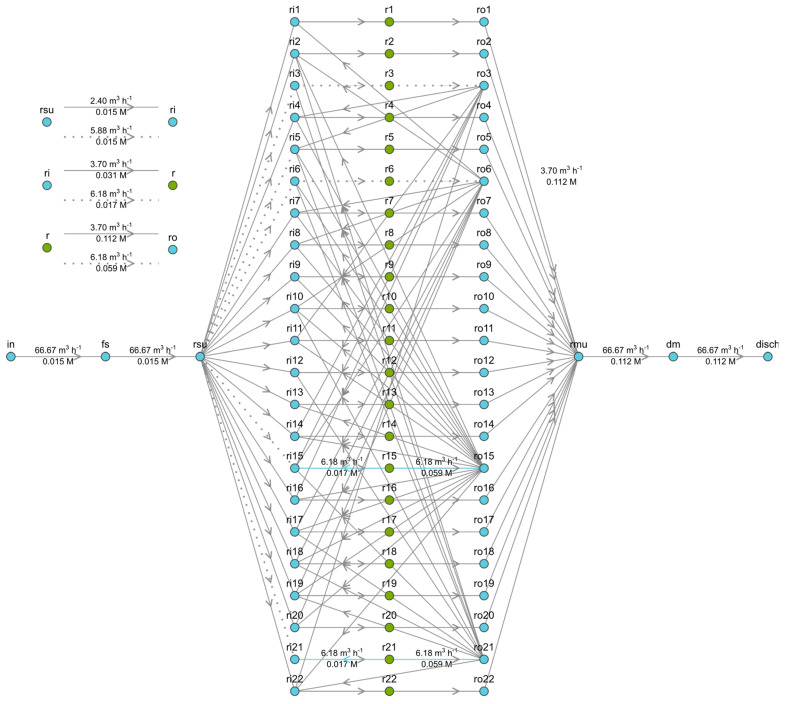
Optimized hydraulic configuration of the LC stream for a modular branch consisting of 22 RUs in S2—case 8. Legend: x = RED unit number; r(x) = RED module x; ri(x) = LC input flow to module x; ro(x) = LC output flow to module x; rsu = splitter unit; rmu = mixer unit; fs = in = LC feed stream; dm = disch = LC discharge.

**Figure 3:**
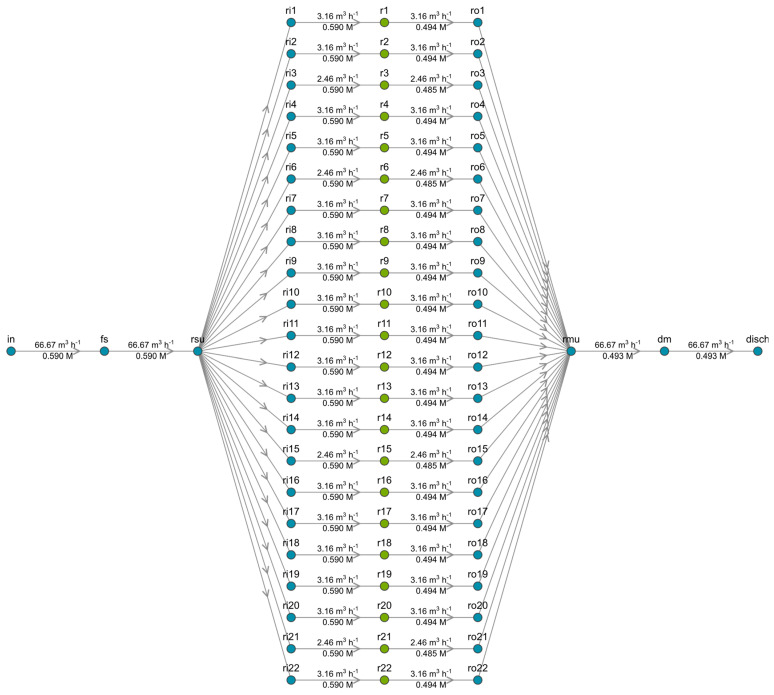
Optimized hydraulic configuration of the HC stream for a modular branch consisting of 22 RUs in S2—case 8. Legend: x = RED unit number; r(x) = RED module x; ri(x) = HC input flow to module x; ro(x) = HC output flow to module x; rsu = splitter unit; rmu = mixer unit; fs = in = HC feed stream; dm = disch = HC discharge.

Modular arrangement diagrams are shown for the LC side in Figure 2, and the corresponding representation for the HC side is shown in Figure 3.

The dilute inflow (LC) is given by the volumetric flow of treated wastewater at the WWTP of Ierapetra and entails a limitation, so the software response includes recirculation streams of the LC to increase the chemical potential utilized and to maximize the objective function defined in the form of NPV.

To maximize the NPV, the solver algorithm has defined four output nodes that are recirculated: ro3, ro6, ro15, and ro21. These branches have a higher flow rate and a lower molarity with respect to the rest of the modules; therefore, it determines that there is still enough practical gradient and recirculates it to the other RED units. This fact is intensified because the LC flow is in deficit given that the optimal ratio is ~0.5 HC:LC [27].

In the case of the HC stream, seawater will be abstracted and conditioned as required, so it will not be a constraint. This avoids the complexity of the hydraulic recirculation system that takes place in the LC.

Looking at the overall balance of matter for NaCl, there is a slight increase in the LC concentration at the outlet of the 22-module parallel branch, from 0.015 M (in) to 0.112 M NaCl (outflow on average).

### 3.3. CAPEX/OPEX Analysis

The annual operating cost (TAC) is mainly determined by the CAPEX, which includes all costs for the RED stack, pumps, and civil and electrical infrastructure, and the OPEX, which includes electricity consumption for pumping, membrane replacement expenses, and operation and maintenance costs.

**Figure 4:**
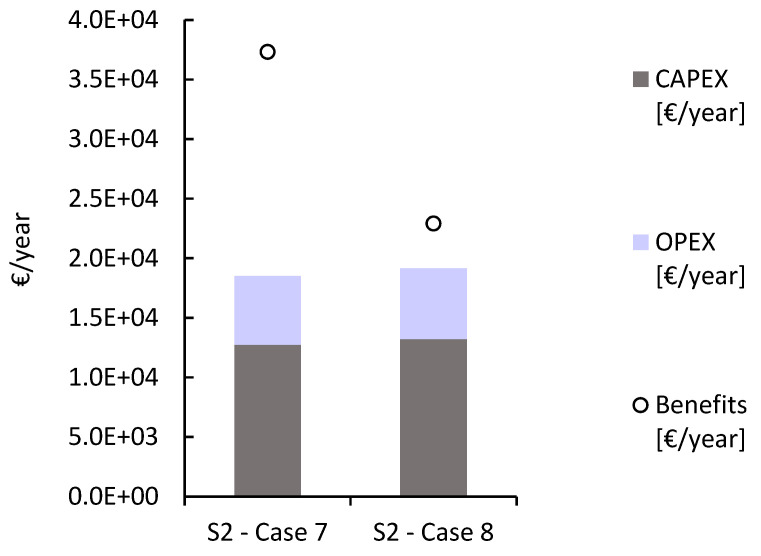
Distribution of annualized capital and operating costs in the Ierapetra WWTP for the following specifications: winter period > average EU electricity price > membrane price 4 EUR/m^2^ (S2—case 7, RU = 53) and 10 EUR/m^2^ (S2—case 8, RU = 22).

Figure 4 shows the fixed costs resulting from the installation of an SGE-RED plant at the Ierapetra wastewater treatment plant under two different equipment investment considerations. The distribution of the total costs in CAPEX and OPEX is not practically affected in the compared cases, representing 69% and 31% of the total annual costs, respectively. This graphical representation displays the impact of the IEM price on the economic benefits of SGE harnessing and the discount of carbon dioxide emission rates.

The benefits increase significantly by lowering the necessary equipment investment per module, which allows the installation of a greater number of RED stacks, resulting in higher profitability.

### 3.4. Economic Comparison of SGE-RED on the Energy Market

The LCOE indicates the ratio of the lifetime costs of building and operating a power plant to the energy that is capable of being generated by that particular facility. This indicator serves to compare the market competitiveness of the electricity generated by renewable energy against conventional alternatives.

**Figure 5:**
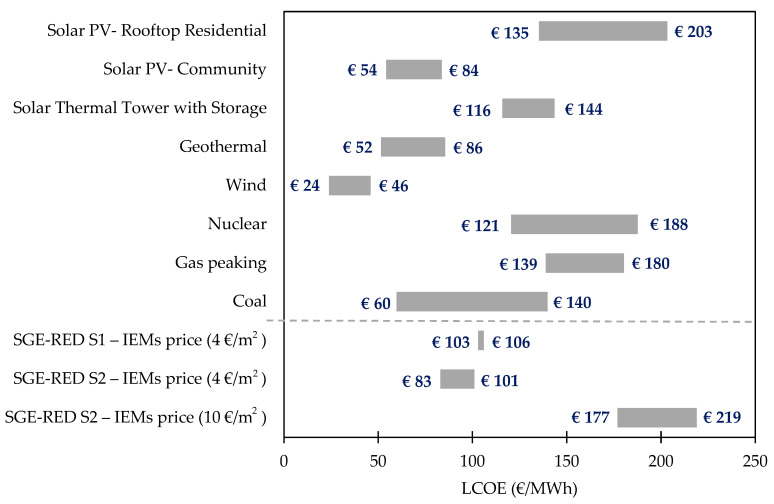
Levelized Cost of Energy for selected renewable and conventional technologies in comparison with SGE-RED harvested in site-specific emplacements S1 and S2 at different membrane prices. Data source: Lazard’s Levelized Cost of Energy Analysis—version 15.0 [45] and optimal simulation results.

Figure 5 presents the LCOE associated with electricity from various renewable and non-renewable sources compared to the optimal results for real sites S1 and S2 at different fixed membrane prices. As confirmed by the graphical representation, SGE-RED would be cost-competitive with conventional alternatives (coal or nuclear) under certain conditions, i.e., at a more affordable core cost of the technology. In the best case studied, SGE-RED S2—IEM price 4 EUR/m^2^, the LCOE would be in the range of 83 EUR/MWh–101 EUR/MWh below gas picking, nuclear power, or PV panels in residential rooftops. A relatively high grid energy tariff compared to the EU average, as reflects the situation of Greece, improves the economic interest of RED by reducing its LCOE.

A reduction in the LCOE index of SGE-RED will make it cost-competitive with other more mature technologies [43]. To this end, IEM development represents one of the main bottlenecks to enhancing net power density. To overcome this drawback, it is urgent to manufacture highly efficient membranes, at an affordable cost, with high selectivity to monovalent ions, and reinforced structure for dimensional stability in scale-up and long-term stable use [44]. LCOE drop will lead to a convergence between the different technologies available on the market. A key factor in further reducing the costs of this technology will be the ability to evolve and reach an industrial scale in order to reduce operating and capital costs.

## 4. Conclusions

The optimal design of up-scaled RED systems for SGE harnessing according to the maximum NPV has been assessed in this study as a function of: (1) site-specific conditions (effluent temperature and flow rate) and (2) economic parameters (membrane and electricity price) by means of two case studies, the WWTPs of Comillas (Spain) and Ierapetra (Greece).

In this context, it has been determined that for the current costs of commercialization of RED equipment (10 EUR/m^2^), feasibility of the Comillas WWTP is subject to the available effluent flow (LC) and its temperature. Thus, the economic balance is positive under the assumption of a more affordable membrane price equivalent to 4 EUR/m^2^. The net reimbursements obtained during the winter season with 29 RUs and generating 7.1 kW are EUR 73 thousand. In summer, the operating units would be 21, generating 5.6 kW and an associated NPV of EUR 60 thousand.

In the Ierapetra WWTP, all simulated conditions are economically profitable, with membrane prices equal to 4 and 10 EUR/m^2^ and national and European electricity cost. It has been found that the ratio of optimal RUs in winter/summer is close to one, leading to the conclusion that there is no over-dimensioning between both seasonal periods of operation.

The design of an industrial RED plant in Ierapetra, considering the current price of electricity in Greece and an up-to-date market cost of membranes of 10 EUR/m^2^, is the most fact-based and therefore objective case study. The results yielded in the operational optimum would result in a profit of EUR 117 thousand operating with 30 RUs in winter and EUR 157 thousand for 32 RUs in summer, and the harnessed SGE would be equivalent to 10.43 kW and 11.96 kW for winter and summer, respectively.

As a general summary, the results achieved for a membrane price of 4 EUR/m^2^ are feasible and promising, considering that in previous studies the break-even price of IEMs was determined at 4.3 EUR/m^2^.

## Figures and Tables

**Table 1:** Definition of the scenarios studied for both sites.

Scenario	S1. Comillas (Spain, ES) ^a^		S2. Ierapetra (Greece, GR) ^b^	
	Flow Rate (m^3^/day)	T (°C)	Flow Rate (m^3^/day)	T (°C)
Winter season	1161	14	1600	18
Summer season	832	20	1600	24

^a^ Atlantic Ocean salinity = 35.5 g/L; Na^+^ 10,770 ppm and Cl^−^ 19,374 ppm [31]. ^b^ Mediterranean Sea salinity = 37.8 g/L; Na^+^ 12,500 ppm and Cl^−^ 22,100 ppm [32].

**Table 2:** Summary of parameters considered for the evaluation of the total annual cost (TAC).

Parameter	Value	Unit	Reference
Electricity price EU *, ep	0.251	EUR/kWh	[39]
Electricity price ES *, ep	0.256	EUR/kWh	[39]
Electricity price GR *, ep	0.339	EUR/kWh	[39]
Carbon price EU, cp	78.16	EUR/ton	[40]
Emission factor ES, ef	0.374	kgCO_2_ eq/kWh	[41]
Emission factor GR, ef	0.813	kgCO_2_ eq/kWh	[41]
Interest rate, r	7.5	%	[42]
Load factor, LF	90	%	[30]
IEM price, cm	4–10	EUR/m^2^	[36,37]
IEM lifetime, LT_m_	15	years	-
Plant lifetime, LT	30	years	-

* Industrial use.

**Table 3:** Characteristics of typical commercial RED modules manufactured by Fumatech BWT GmbH^®^ (Bietigheim-Bissingen, Germany).

Parameter	Value	Unit
Total IEM area stack, A_m,r_	2 × 0.175	m^2^
Cell pairs, N_cp_	1000	-
Spacer thickness, δ_sp_	270	µm
Width, b × length, L	0.456 × 0.383	m^2^

**Table 4:** Simulation results of the cases considered and their specific conditions.

		Working Conditions			Optimal Results		
	Case	Scenario	Electricity Price (EUR/kWh)	IEM Price (EUR/m^2^)	RU	NPV (EUR)	TNP (kW)
S1	1	Winter Season	ES	4	29	66,878	7.12
	2			10	0	‒	‒
	3	Summer season	ES	4	21	55,453	5.62
	4			10	0	‒	‒
S2	5	Winter Season	GR	4	58	320,786	14.45
	6			10	30	116,501	10.43
	7		EU	4	53	204,720	13.95
	8			10	22	40,723	8.50
	9	Summer season	GR	4	58	361,535	15.57
	10			10	32	156,839	11.96
	11		EU	4	54	237,634	15.20
	12			10	24	69,302	10.03

## Data Availability

Not applicable.

## Nomenclature

**Nomenclature**	**Description**	**Units**
A_m,r_	Total IEM area stack	m^2^
b	Width	m
CAPEX	Capital expenses	EUR/year
cm	IEM price	EUR/m^2^
cp	Carbon price	EUR/tCO_2e_
CRF	Capital recovery factor	-
ef	Emission factor	kgCO_2 eq_/kWh
ep	Electricity price	EUR/kWh
L	Length	m
LCOE	Levelized Cost of Energy	EUR/MWh
LF	Load factor	%
LT	Plant lifetime	years
LT_m_	IEM lifetime	years
N_cp_	Cell pairs	-
NP_r_	Net power per RED unit	kW
NPV	Net present value	EUR
OPEX	Operational expenses	EUR/year
Q_LC_	LC flow rate	m^3^/h
r	Interest rate	%
TAC	Total annual cost	EUR/year
TNP	Total net power	kW
TNSE	Total Net Specific Energy	kWh/m^3^
δ_sp_	Spacer thickness	µm

## Abbreviations

**Abbreviations**	**Description**
ES	Spain
GDP	Generalized disjunctive program
GR	Greece
HC	High-concentrated stream
IEM	Ion exchange membrane
LC	Low-concentrated stream
p.e.	Population equivalent
RED	Reverse electrodialysis
RU	RED units
S1	Site 1, Comillas
S2	Site 2, Ierapetra
SGE	Salinity gradient energy
TRL	Technology Readiness Level
UWWTP	Urban wastewater treatment plant
WWTP	Wastewater treatment plant
